# Comparing free-space and fiber-coupled detectors for Fabry–Pérot-based all-optical photoacoustic tomography

**DOI:** 10.1117/1.JBO.27.4.046001

**Published:** 2022-04-05

**Authors:** Jakub Czuchnowski, Robert Prevedel

**Affiliations:** a European Molecular Biology Laboratory (EMBL), Cell Biology and Biophysics Unit, Heidelberg, Germany; b Collaboration for Joint PhD Degree beween EMBL and Heidelberg University, Faculty of Biosciences, Heidelberg, Germany

**Keywords:** photoacoustics, tomography, imaging, detectors, Fabry–Perot, fiber applications

## Abstract

**Significance:**

Highly sensitive detection is crucial for all-optical photoacoustic (PA) imaging. However, free-space optical detectors are prone to optical aberrations, which can degrade the pressure sensitivity and result in deteriorated image quality. While spatial mode-filtering has been proposed to alleviate these problems in Fabry–Pérot-based pressure sensors, their real functional advantage has never been properly investigated.

**Aim:**

We rigorously and quantitatively compare the performance of free-space and fiber-coupled detectors for Fabry–Pérot-based pressure sensors.

**Approach:**

We develop and characterize a quantitative correlative setup capable of simultaneous PA imaging using a free space and a fiber-coupled detector.

**Results:**

We found that fiber-coupled detectors are superior in terms of both signal level and image quality in realistic all-optical PA tomography settings.

**Conclusions:**

Our study has important practical implications in the field of PA imaging, as for most applications and implementations fiber-coupled detectors are relatively easy to employ since they do not require modifications to the core of the system but only to the peripherally located detector.

## 1 Introduction

All-optical photoacoustic (PA) tomography is an emerging alternative to classical piezoelectric approaches.^1^ Multiple optical detector types and geometries are constantly being developed and improved with the overarching aim of matching the detection sensitivity of piezoelectric systems. Among them, Fabry–Pérot interferometer (FPI) sensors are particularly promising, as they combine the ability to measure acoustic waves with high spatial resolution and pressure sensitivity. For this application, a pressure-sensitive FP device is formed by sandwiching a thin layer (10 to 100  μm) of elastomer (e.g., Parylene C) between two dichroic mirrors. This optical resonator has then the ability to elastically deform under pressure, modulating the FP interferometer’s transfer function (ITF), which is a function of the cavity thickness. The sensor is then interrogated by tuning the laser wavelength to the point of maximum slope on the ITF (so-called bias wavelength) which translates the acoustic (pressure) waves into a modulation of an optical (interference) signal. In practice, this approach has allowed acoustic sensing in the range of 100 to $10^{6}\;\mathrm{Pa}$ with a very broadband frequency response (bandwidth ∼0.1 to 40 MHz).^2^^,^^3^

As optical devices, FPIs are sensitive to light beam aberrations, which can have detrimental effects on their performance under certain conditions. Among different types of optical sensors, FP cavities are especially sensitive to aberrations as their sensing principle is dependent on the high spatial uniformity of the light beam to facilitate efficient interference.^4^ It was previously shown that both beam and cavity aberrations,^5^ surface roughness^6^ as well as mirror non-parallelism^4^^,^^7^ can lead to severe deterioration of the optical sensitivity of the FPI and that this loss can be partially recovered by the use of aberration correction techniques including adaptive optics.^8^ An alternative method of aberration correction is based on spatial-mode-filtering^4^^,^^5^^,^^9^ where a single-mode fiber^5^^,^^9^ or an optical pinhole^4^ is used to reject part of the interrogation light to improve the measurement sensitivity. This effect can be explained using different frameworks, either as rejecting more divergent components of the beam which carry lower contrast interference fringes^4^ or removing higher-order spatial modes of the beam which carry spectrally shifted interference fringes which lower the contrast (and hence sensitivity) of the ITF.^5^ Experimentally, significant improvements of sensitivity by mode-filtering were shown with the use of a single-mode fiber-coupled detector (FCD).^5^^,^^9^ Although FCDs are commonly used in the community,^10^^–^^12^ their advantages and disadvantages to free space detectors (FSDs) were never directly compared in realistic, experimental conditions. Hence, the real functional advantage of FCDs remained unclear, since important factors such as the effective sensitivity gain as well as expected power losses in FCDs were not previously quantified. In this work, we, therefore, aimed to rigorously compare FCDs and FSDs while taking into account differences in photodetector working points, fiber-coupling efficiency as well as the frequency response of the photodiodes. We found that an FCD is capable of not only significantly improving the optical sensitivity, but also the ultrasound signal level, both of which ultimately translate to improvements in PA image quality. Importantly, FCDs can achieve these gains with only moderate losses in the transmitted power as compared to FSDs. Taken all together, these are strong arguments for the use of FCDs in FP-based PA sensing and imaging experiments.

## 2 Materials and Methods

### 2.1 Experimental Design

The experimental system consists of a fiber-coupled tunable external cavity laser (Venturi TLB-8800, Spectra-Physics) collimated by lens L1 (F240APC-1550, Thorlabs) and illuminating a custom made FPI via a 70:30 (R:T) beamsplitter (BS024, Thorlabs), a set of XY galvanometric mirrors (GVS012/M, Thorlabs) and a scan lens (2x# 47-319, Edmund). The back-reflected light is split on the BS and a part of it is focused by lens L2 (2xAC254-050-C, Thorlabs) onto an amplified photodetector (PDA05CF2, Thorlabs) referred further as the FSD. The other part of the light is back-coupled into the laser output fiber (SMF-28e, NA 0.14, FS) and redirected via a circulator (FM-PICIR-3-X-X, FS) to the FCD that consists of lenses L3 (F240APC-1550, Thorlabs) and L4 (2xAC254-050-C, Thorlabs) focusing the light onto another amplified photodetector of the same type (PDA05CF2, Thorlabs).

This design using back-coupling into the same fiber with redirection using a circulator simplifies the optical path and allows for high coupling efficiency as the beam can be freely resized to match the PA measurement requirements imposed by the FPI.

### 2.2 Photoacoustic Imaging and Image Reconstruction

The wire phantom was prepared by suspending a 30  μm nylon surgical suture (NYLON ZO030590) on a custom three-dimensional (3D) printed scaffold and submerging it in a water bath.

This work was done in accordance with the European Communities Council Directive (2010/63/EU) and all procedures described were approved by EMBL’s committee for animal welfare and institutional animal care and use. Experiments were performed using C57Bl6/j transgenic mice from EMBL Heidelberg core colonies. An aqueous gel was inserted between the skin and the FPI sensor head to facilitate acoustic coupling. For imaging, mice were anesthetized with isoflurane (2% in oxygen, Harvard Apparatus). Body temperature was kept constant throughout the experiments by the use of a small animal physiological monitoring system (ST2 75-1500, Harvard Apparatus), and eyes were covered with ointment to prevent drying. The diameter of the excitation beam incident on the skin surface was ≈1.5  cm, and the fluence was $\approx 1\;\mathrm{mJ}\;{\mathrm{cm}}^{-2}$ and was thus below the safe maximum permissible exposure for the skin.^13^

The FPI used in this study closely resembles previously published FPI designs^2^ and uses dielectric mirrors with 98% reflectivity between 1500 and 1600 nm on a slightly wedged polymethyl methacrylate backing. A ∼20  μm Parylene C spacer is then deposited in-between the mirrors using vapor deposition. The maximum scan area was $8\times 8\;{\mathrm{mm}}^{2}$ and a typical scan acquired ∼6000 waveforms each comprising over 1000 time points (sampling rate 125 MHz, ATS9440-128M, AlazarTech). The image acquisition time was ≈10  min and was limited by the response time of the interrogation laser. The diameter of the focused interrogation laser beam was 92  μm, which, to a first approximation, defines the acoustic element size. As fiber coupling might cause substantial losses in the transmitted power, we compared the transmitted power between the FCD and the FSD and quantified it to be on average 47±35% (mean ±2σ, n=6561 point on the FPI). The large variation in the transmitted power probably stems from imperfections of the optical setup (limited telecentricity of the scan lens combined with a non-conjugated galvo system) which could in principle be improved for higher power transmission.

All images shown are based on averaging three subsequent excitation pulses at each scan position. Following the acquisition of the PA signals, the following protocol was used to reconstruct and display the images: (1) To correct for the effects of photodiode working point, the signals were normalized by the power incident on the PD at each scan position which was acquired during the characterization. (2) For the mouse image, the recorded PA signals were interpolated onto three times finer spatial grid. (3) The tissue sound speed was estimated using an autofocus method.^14^ (4) A 3D image was then reconstructed from the interpolated PA signals using a time-reversal-based algorithm^15^ for the mouse image and a back-projection algorithm^16^ for the wire phantom with the sound speed obtained in step (4) as an input parameter. The image reconstruction was implemented using k-Wave, an open-source Matlab toolbox.^16^

### 2.3 Quantification of FPI Optical Sensitivity

We use an approach based on fitting of the Psuedo–Voigt function

$$V_{p}(x)=\eta \cdot L(x,f_{L})+(1-\eta )\cdot G(x,f_{G}),$$ (1)

where $L(x,f_{L})$ is a Lorentzian with $f_{L}$ being the full width at half maximum (FWHM) parameter of the Lorentzian, $G(x,f_{G})$ is a Gaussian with $f_{G}$ being the FWHM parameter of the Gaussian (see Refs. 5 and 12 for details). We calculate the normalized optical sensitivity from the fit according to our previous work.^5^

### 2.4 Quantification of PA Image Quality

We use three previously described and routinely used image metrics^14^ to compare PA image quality between the FSD and FCD detectors:

1. The Brenner gradient is given by
  $$Q_{\mathrm{Brenner}}=\sum_{x,y}(I(x+2,y)-I(x,y){)}^{2}+(I(x,y+2)-I(x,y){)}^{2},$$ (2)
  where I(x,y) are the pixel values of the image at point (x,y).
2. The Tenenbaum gradient is given by
  $$Q_{\mathrm{Tenenbaum}}=\sum_{x,y}(s*I(x,y){)}^{2}+(s^{T}*I(x,y){)}^{2},$$ (3)
  where s is the Sobel operator, stated as
  $$s=(\begin{array}{ccc} -1 & 0 & 1\\ -2 & 0 & 2\\ -1 & 0 & 1 \end{array}).$$ (4)
3. And normalized variance is
  $$Q_{\mathrm{NormVariance}}=\frac{1}{\left\langle I\right\rangle }\underset{x,y}{\sum }(I(x,y)-\langle I\rangle {)}^{2},$$ (5)
  where ⟨I⟩ is the mean intensity of the image.

## 3 Results

### 3.1 Correlative FPI Characterization Using Free-Space and Fiber-Coupled Detectors

**Fig. 1:**
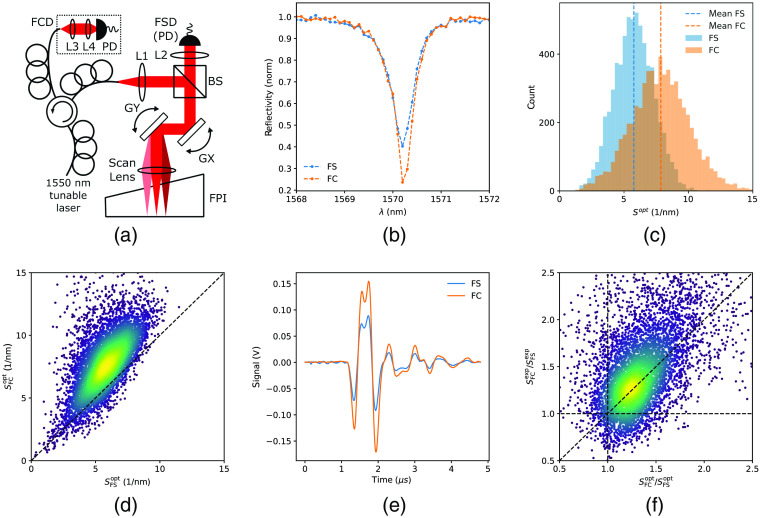
(a) Schematic of the PAT system for correlative imaging using an FSD and FCD. PD, photodiode; Lx, lens x; GX, GY, galvo mirrors; BS, non-polarizing beamsplitter; FSD, free-space detector; FCD, fiber-coupled detector. (b) Comparison of representative free-space and fiber-coupled ITFs showing an increase in visibility in the fiber-coupled condition. (c) Comparison of the free-space and fiber-coupled normalized optical sensitivity showing an increase in visibility in the fiber-coupled condition. (d) Point-by-point comparison of the normalized optical sensitivity shows a point-wise improvement in sensitivity for the majority of spots on the FPI. (e) Exemplary ultrasound waveform recorded by the FSD and FCD. (f) Point-wise comparison between the characterized improvement in optical sensitivity and the measured improvement in ultrasound signal level.

To meticulously study the previously suggested^5^^,^^9^ differences between FSD and FCD, we implemented a custom correlative FP-based photoacoustic tomography (PAT) setup [Fig. 1(a)]. We performed simultaneous characterization of the FPI using both an FSD and FCD across ∼6500 scan points over an 8×8  mm sensor surface. We found that the FCD significantly improves the ITF in visibility [Fig. 1(b)] which is a strong indicator for increased optical sensitivity. We then proceeded to measure the normalized optical sensitivity^5^ across the surface of the FPI and observed that the FCD indeed shows an increase in sensitivity [Fig. 1(c)], which translates to an overall increase in the order of ∼30% across the whole FPI sensor. To investigate this increase, further, we analyzed the data in a point-wise manner and observed that the increase in sensitivity is uniform with almost all characterized points exhibiting a higher sensitivity with the FCD [Fig. 1(d)].

To ascertain that the apparent increase in optically measured sensitivity actually translates to improved ultrasound sensing capabilities, we performed correlative ultrasound measurements using our system. We observed that the measured ultrasound amplitude is higher by 43±0.11% (mean $\pm 2\bar{\sigma }$ for n≈4400 scan positions) for the FCD [Fig. 1(e)], which is in agreement with the increased optical sensitivity of the FCD. However, it is important to note that differences in the working point between the FCD and FSD need to be taken into account for proper comparison between the conditions as the ultrasound amplitude is directly proportional to the direct current (DC) level of the photodetector. This effect can be removed by normalizing the measured signals by the working point known from the FPI characterization. We, therefore, also analyzed the data in a point-wise manner by plotting the normalized experimentally measured signal improvement ($S_{\mathrm{FC}}^{\exp}/S_{\mathrm{FS}}^{\exp}$) against the increase in normalized optical sensitivity ($S_{\mathrm{FC}}^{\mathrm{opt}}/S_{\mathrm{FS}}^{\mathrm{opt}}$) and we observe that the two are in good agreement [Fig. 1(f)], corroborating the observation that the FCD shows an increase in effective sensitivity. Despite our efforts to accurately quantify both the optical as well as acoustic gains, there are still off-diagonal outliers present in the data. These presumably stem either from small distortions in the transfer function due to the wavelength dependence of the BS splitting ratio that affect quantifying the optical sensitivity or from small differences in the frequency response of the photodiodes employed that affect quantifying the ultrasound sensitivity.

### 3.2 Correlative Imaging Comparing Free-Space and Fiber-Coupled Detectors

**Fig. 2:**
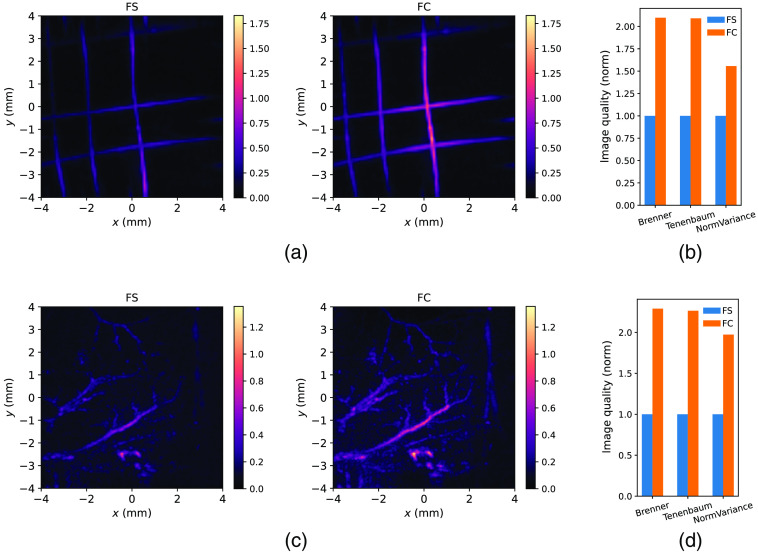
(a) Experimental PAT image of a wire phantom using FCD and FSD. Intensity is normalized to the brightest pixel in the free-space image and both images use the same color scale. The images were up-sampled to a three times finer spatial grid for better visualization. (b) and (d) Quantification of image quality for panels (a) and (d) using common image quality metrics. (c) Experimental PAT images of a live mouse lower back at 600 nm excitation wavelength using fiber-coupled and free-space detectors. (c) and (d) Intensity is normalized to the brightest pixel in the free-space image and both images use the same color scale.

Having characterized the FPI-based system both optically and using ultrasound sources. we went on to characterize the PA imaging properties of the FCD and FSD. We performed PA imaging of a wire phantom and observed that the reconstructed image intensity is higher for the FCD [Fig. 2(a)] which is consistent with the characterization results. Here also, the PA waveforms were normalized to the working point of the photodiode to remove the effect of detector differences from the PA signal amplitude. We quantified the increase in image quality by calculating commonly used image quality metrics^14^ and show that for all metrics there is a significant improvement in image quality when using the FCD [Fig. 2(b)].

We further compared the performance of FC and FS detectors by performing *in vivo* mouse vasculature imaging experiments. We acquired PAT data from the lower back area using 600 nm excitation to visualize the vasculature in a label-free manner. We observed that also in this case the FCD provides a significantly better image quality [Fig. 2(c)] which can also be quantified using appropriate metrics [Fig. 2(d)]. This corroborates the superiority of the FCD for both phantoms as well as *in vivo* imaging in FPI-based PAT.

## 4 Discussion

We have experimentally demonstrated that mode-filtering with the use of FCDs is capable of significantly improving the sensitivity of FPI-based PAT. This finding has important practical implications as for most applications and implementations fiber-coupled detectors are relatively easy to employ as they do not require modifications to the core of the system but only to the peripherally located detector. We would like to highlight that the obtainable improvements are still dependent on the FPI properties such as thickness, interrogation spot size, and surface quality of the FPI. Based on our observations, however, for FPIs relevance of PA imaging, we expect an overall improvement in the same order as reported here.

Additionally, we note that careful optical design is required because experimentally induced losses in fiber coupling may be significant and potentially disadvantageous overusing FSD. We note that further improvements to the sensitivity can be obtained in principle by combining fiber-based mode filtering with active wavefront modulation approaches (as previously suggested in Ref. 5).

To achieve this, however, several technical challenges need to be overcome in the future, especially on the optical engineering side to increase the beam stability in the system allowing for efficient back-coupling into the fiber in conjunction with active wavefront shaping.

## Code, Data, and Materials Availability

The datasets generated during and/or analyzed during this current study are available from the corresponding author on reasonable request.
